# Are Dietary Intakes Related to Obesity in Children?

**DOI:** 10.3889/oamjms.2016.045

**Published:** 2016-03-22

**Authors:** Dimitrios Papandreou, Kali Makedou, Areti Zormpa, Maria Karampola, Anastasia Ioannou, Areti Hitoglou-Makedou

**Affiliations:** 1 *Department of Natural Science and Public Health, Zayed University, Abu Dhabi, United Arab Emirates*; 2 *Laboratory of Biological Chemistry, Medical School, Aristotle University of Thessaloniki, Thessaloniki, Greece*; 3 *Laboratory of Lipids and Cardiovascular Disease Prevention from Childhood, 2nd Pediatric Department, AHEPA University Hospital, Thessaloniki, Greece*

**Keywords:** dietary intake, obesity, children, Greece, dietary iron deficiency

## Abstract

**AIM:**

The purpose of this study was to report obesity status and identify any dietary substances that may be related to obesity in healthy school children from Northern Greece.

**METHODS:**

Four hundred and twenty-five (n = 425) children were randomly selected to participate in the study. A 24-h recall of three days (two weekdays and one weekend day) was used to analyze the dietary data of the subjects.

**RESULTS:**

Out of 425 subjects, 146 (34.3%) of them were found to be overweight and obese. Energy, protein, carbohydrate and thiamin intake was statistically positively correlated with obesity while dietary iron intake was statistically negatively correlated with obesity. Multivariate logistic regression analysis showed that the children with dietary iron deficiency were 1.128 (95% CI: 0.002, 0.161 P < 0.031) times more likely of being obese compared to the normal group after adjustment for energy intake.

**CONCLUSIONS:**

Although most of the dietary intakes of our subjects were adequate, special consideration should be given to energy, carbohydrate, protein, and sugar and iron intake especially and its relation to obesity. Furthermore, additional studies are required to investigate any possible relation of low dietary iron consumption and obesity.

## Introduction

Pediatric obesity is increasing worldwide and it is associated with an increased risk of obesity later in life [1]. Excess weight in children has been found to be related to many cardiometabolic risk factors such metabolic syndrome, type 2 diabetes, hypertension, insulin resistance, metabolic syndrome and dyslipidemia [2]. The primary cause of pediatric obesity is not well understood. It is a multidisciplinary disease that involves genetics, environment and lifestyle factors and of them that have been found to play a significant role is a dietary pattern [3]. Childhood obesity has been connected to the unhealthy dietary patterns (fats, red meat, sugars, and iron deficiency) by many researchers [4]. On the other side vegetable proteins intakes have recently found that may play a role in preventing obesity among European adolescents [5].

It is also well known that iron deficiency is higher in obese children compared to normal ones [6]. It has been documented preadolescents with high body fat levels tend to have higher levels of iron deficiency [7]. Data regarding males and females have shown that there is no difference between of them and iron deficiency [6]. Dietary iron intake was also found to have no difference between obese and no obese children [7].

In addition, the Mediterranean diet that has been used to describe the good dietary habits of people in Greece and other Mediterranean countries [8] has been gradually abandoned by people and especially children [9]. Dietary intake data related to obesity has been reported by neighbor countries [10, 11] and in whole Europe [12]. Recent information from European data on nutrient intake has revealed that SFA and salt were higher in children and adolescents [13]. In the same study, the consumption of folate, vitamin D and iodine was found to be lower than 55% of the recommendations [13].

However, in Greece [14, 15] data regarding obesity in childhood and its relation to dietary and nutritional status is limited.

As a result, it has become important to understand children’s dietary patterns and develop appropriate methods to nurture healthy eating habits among them. The aim of the present study was to report the prevalence of obesity and data of children, to evaluate the nutrient intake of children and adolescents by analyzing a 3-day 24-hour recall dietary and to find any possible associations between dietary intakes and obesity.

## Methods and Subjects

The study was conducted in the framework of INTERREG IIIa and initially included 893 children, 445 boys and 438 girls (mean age 10.5, range 9-12 years), from rural areas of prefectures of Thessaloniki (the second biggest city in Greece) Kilkis and Serres, of Northern Greece, that were chosen according to the population size and the location (distance from Thessaloniki). The total population of these areas are 920,000 thousand people (Thessaloniki 800,000, Kilkis 50,000 and Serres 70,000). Some of the schools that participated are proximate to Thessaloniki, whereas some of them are situated near the northern borders of Greece.

These figures are the most valid based on Greek statistics. For the sample size, the statistical error was 3%. The selection of the subjects was performed randomly from schools that accepted to participate in the study. Out of 893 questionnaires distributed, only 425 children returned them filled in and finally accepted to participate in the study, 197 boys and 228 girls. Children with diabetes, cardiovascular, liver, renal and any other serious disorder were excluded from the study. The aim of the study was clearly explained to the children and parents orally and a written consent form was signed by the parents of the children. The study was approved by the ethical committee of Aristotle University of Thessaloniki, Greece.

All measurements took place in the school setting after 10-hour fasting. Height was measured with a Raven Minimeter (Raven Equipment Limited, Essex, United Kingdom), with no shoes on, to the nearest 0.1cm. Weight was measured with a Seca weighing machine [Seca, Hanover, MD], without shoes, to the nearest 100 grams. BMI was calculated and children were classified based on the age and sex cut-off points for BMI, based on the International Obesity Task Force recommendations [16]. Tanner stage was evaluated in all children by one well-trained and experienced pediatrician in each prefecture.

Dietary intake data of 3 days (2 weekdays and 1 weekend), was collected by a registered dietitian using a 3-day dietary recall technique. This particular technique of the 3-day, 24h recall, has been previously evaluated successfully in children aged above 9 years old [17]. The result from the three 24h recalls was reported and analyzed as mean dietary values. The family members together with the children answered the questionnaire. The Nutritionist V diet analysis software [version 2.1, 1999, First Databank, San Bruno, CA, USA], was used to analyze the dietary data. Additional Greek foods and recipes have been included in the software as previously described [18].

### 

#### Statistics

The statistical package IBM SPSS Statistics 20.0 (IBM Corp.) was used for the statistical analysis of data. The Kolmogorov–Smirnov test was used in order to test the normality of distribution of values. Values were expressed as median (range). For non-parametric comparisons between two independent groups, Mann-Whitney U-test was used. For non-parametric correlations between independent parameters Spearman’s correlation coefficient was calculated. Scatter plots were created demonstrating significant correlations. Finally, logistic regression analysis was performed in order to investigate the possible dependence of obesity to dietary constituents. Statistical significance was considered at *P* < 0.05.

## Results

The prevalence of overweight and obesity together was 34.3% (data not presented). No differences were observed in children between tanner stages (data not shown). The anthropometric and dietary characteristics based on gender are presented in Table 1. Dietary fiber intake was found to be statistically higher in girls compared to boys.

**Table 1:** Anthropometric and dietary characteristics of the subjects based on gender (N=425)

Characteristic	Boys N = 197 Median (min-max)	Girls N = 228 Median (min-max)	*P*
Age (years)	11 (9-12)	10 (9-12)	0.384
Height (cm)	143 (123-173)	144 (120-170)	0.212
Weight (kg)	39 (19-83)	39.5 (21-77)	0.517
BMI (kg/m2)	18.8 (18.2-32.7)	18.6 (17.5-29.3)	0.565
Energy (Kcal)	2053 (1131-3925)	2014 (1190-4485)	0.406
Protein (g)	80.4 (21-160)	81.4 (22-164)	0.438
Carbohydrates (g)	212 (107-401)	221 (105-443)	0.193
Total Fat (g)	96 (27-230)	98 (35-214)	0.947
Caffeine (μg)	5.7 (1-81)	6.9 (1-95)	0.831
Fiber (g)	12 (2-35)	13 (2-37)	0.020*
Sugar (g)	10 (5-48)	17 (8-51)	0.097
Vit A (IU)	3545 (386-16537)	3591 (624-22915)	0.582
Vit D (mg)	143 (10-446)	142 (15-419)	0.931
Vit E (mg)	8 (2-93)	9 (3-48)	0.462
Vit K (mg)	134 (74-394)	154 (88-542)	0.561
Vit C (mg)	71.2 (19-422)	82.5 (14.7-378)	0.226
Thiamin (mg)	2 (0.3-4.1)	2.2 (0.7-5.2)	0.681
Riboflavin (mg)	2.1 (0.7-6.1)	2.4 (0.8-6.4)	0.810
Niacin (mg)	19.2 (3.1-46.2)	20.1 (3.8-45.1)	0.416
Vit B6 (mg)	1.7 (0.4-4.4)	1.65 (0.6-4.2)	0.421
Folic Acid (μg)	431 (105-629)	401 (112-588)	0.674
Vit B12 (μg)	4.5 (0.16-17.2)	4 (1-19)	0.281
Calcium (mg)	1124 (198-1565)	1129 (140-2180)	0.541
Magnesium (mg)	222 (79-455)	232 (22-438)	0.282
Potassium (mg)	2295 (820-4190)	2300 (883-5120)	0.307
Sodium (mg)	2326 (240-7200)	2537 (291-6130)	0.408
Iron (mg)	13 (2-25)	13.7 (1-34)	0.289
Zinc (mg)	9 (1-20)	8.1 (3-19)	0.896

* Statistically significant difference *P*<0.05.

Abrev: BMI: Body Mass Index, Vit: vitamin

Table 2 analyzes the anthropometric and dietary characteristics based on normal and overweight/obese status of subjects. The overweight/obese group was statistically significantly found to consume higher levels of energy intake, carbohydrates, sugar, sodium and lower levels of dietary thiamin, folic acid, iron, and zinc, compared to the normal group.

**Table 2:** Characteristics of normal and overweight/obese subjects (N=425)

Characteristic	NORMAL N = 279 Median (min-max)	OW + OB N = 146 Median (min-max)	*P*
Age (years)	10.5 (9-11.5)	11 (9.5-12)	0.677
BMI (kg/m2)	18.9 (18.2-21.2)	23.4 (19.3-32.7)	0.001*
Energy (Kcal)	1971 (1150-3451)	2344 (1395-4880)	0.033*
Protein (g)	77 (21-164)	82.5 (44-179)	0.042*
Carbohydrates (g)	209 (74-443)	232 (77-463)	0.008*
FAT (g)	95.8 (29-185)	100 (35-210)	0.124
Caffeine (μg)	5.8 (2-14.6)	4.7 (1-139)	0.641
Fiber (g)	13 (3-26)	12 (2.4-21)	0.812
Sugar (g)	8.2 (4.1-14.2)	17 (4.1-43)	0.002*
Vit A (IU)	3537 (852-6521)	3612 (799-6321)	0.574
Vit D (mg)	139 (21-412)	152 (26-441)	0.159
Vit E (mg)	8 (2-63)	8.9 (4-54)	0.300
Vit K (mg)	129 (61-408)	139 (58-398)	0.247
Vit C (mg)	80 (4-422)	76 (9.4-365)	0.778
Thiamin (mg)	2.1 (0.5-4)	1.5 (0.3-4.3)	0.007*
Riboflavin (mg)	2.2 (0.4-8.9)	2.1 (0.5-9.1)	0.063
Niacin (mg)	19.2 (3-42)	16.4 (3.4-45.2)	0.022
Vit B6 (mg)	1.64 (0.4-4.1)	1.81 (0.6-4.2)	0.172
Folic Acid (μg)	342 (54-807)	307 (131-788)	0.024*
Vit B12 (μg)	4 (0.2-22)	4.5 (1-30)	0.292
Calcium (mg)	1105 (307-2421)	1187 (312-2641)	0.110
Mg (mg)	222 (44-412)	241 (81-438)	0.144
Potassium (mg)	2297 (820-5076)	2313 (839-5845)	0.077
Sodium (mg)	2317 (285-7211)	2495 (2110-7824)	0.037*
Iron (mg)	12 (2.4-25)	9 (19-20)	0.004*
Zinc (mg)	9.3 (1-22)	8.1 (3-18)	0.020*

* Statistically significant difference *P*<0.05.

Abrev: OW = overweight, OB = obesity, BMI = body mass index, Vit: vitamin

Energy intake, protein, carbohydrates, thiamin, was found to present a statistical, significant positive correlation with BMI while zinc and iron a statistically negatively correlation, respectively (Table 3).

**Table 3:** Spearman’s coefficients between BMI and dietary variables (N=425)

Variables	rho
Energy (Kcal)	0.018
Protein (g)	0.102
Carbohydrates (g)	0.022
Sugar (g)	0.136
Thiamine (mg)	0.099
Folic Acid (μg)	0.091
Sodium (mg)	0.123
Iron (mg) Zinc (mg)	-0.108 -0.291

* *P*<0.05.

Abrev: BMI=Body, Mass Index

Tables 4 and 5 show the Odds Ratio between obesity and dietary variables before and after adjustment of energy intake. Multivariate logistic regression analysis showed that children with dietary iron deficient was 1.128 (95% CI: 0.002, 0.161; P<0.031) times more likely of being obese compared to the normal group after an adjustment of energy intake (Table 5).

**Table 4:** Crude Logistic regression analysis of different nutrients to predict obesity (N=425)

Variables	Beta (OR)	95% CI	*P*
Energy (Kcal)	0.050	-0.02, 0.01	0.681
Protein (g)	0.017	-0.04, 0.03	0.871
Carbohydrates (g)	0.146	0.01, 0.191	0.170
Iron (mg)	1.141	0.002, 0.171	0.007*
Thiamine (mg)	0.018	-0.050, 0.071	0.759
Zinc (mg)	0.044	-0.006, 0.007	0.432

* *P*<0.05

**Table 5:** Logistic regression analysis of different nutrients to predict obesity (N=425)

Variables	Beta (OR)**	95% CI	*P*
Protein (g)	0.011	-0.04, 0.029	0.668
Carbohydrates (g)	0.129	0.012, 0.172	0.341
Iron (mg)	1.128	0.002, 0.161	0.031*
Thiamine (mg)	0.018	-0.040, 0.059	0.655
Zinc (mg)	0.029	-0.002, 0.004	0.089

* *P*<0.05.

** Adjusted for age, height and energy intake.

## Discussion

The prevalence of overweight and obesity in our study was 34.3%. The results of the present study are lower compared with a study from Alexandroupoli (North-East Greece) that reported prevalence rates of overweight and obesity (40.6 percent) in 709 children [19]. Similar results were also published in a nationwide study, where the authors examined 18,045 children and reported higher levels of overweight and obesity (86.7 percent) compared to our data. However, the data lacks the methodology on weight and height since the entire data recorder was self-reported [20]. In addition, our data showed lower prevalence rates compared to the GRECO study [13]. In this study, the authors examined 4,786 children aged 10-12y and found that the prevalence of OW and OB was 29.9% and 12.9%, while in girls 29.2% and 10.6%, respectively.

Compared to other countries from Europe, the prevalence rates of our study sample are higher. Countries such as Denmark, Finland, Norway and Sweden have reported the prevalence of overweight and obesity between 10 and 15 percent, whereas the Czech Republic, Estonia, Latvia and Russia have reported prevalence rates <10 percent [21]. In addition, recent data from the European WHO [22] has shown that the prevalence rates of OW and OB together, in Mediterranean countries (Spain, Republic of Macedonia, Italy) using the IOTF cut of points, were 41.5 %, 27.4% and 40.4% for boys and 33.5 %, 26.2% and 33.9 for girls, respectively. Moreover, the prevalence of OW/OB from our neighboring country, Turkey [23] revealed rates around 37.6%, which are highly comparable to our results.

The most important finding in our study was that the dietary iron intake consumed by the overweight/obese group was significantly associated with obesity. The overweight/obese group consumed a quantity of 9 mg/d compared with the normal group (12 mg/d). Low iron status in obese children could result from a combination of nutritional and functional iron deficiency. The literature review data is controversial. Results from a Sweden study of 142 children aged 6-14y revealed no association between obesity and dietary iron intake [7]. In contrast, other studies have shown that obesity may contribute to dietary iron deficiency [24, 25]. An explanation to that may be that the peptide hepcidin, which produced in the liver and adipose tissue, is a key regulator of iron homeostasis. Hepcidin expression is increased in chronic inflammation and obesity and may contribute to the increased prevalence of iron deficiency observed in overweight populations [24, 25]. Furthermore, weight loss in obese children has been found to lead to a decrease in serum hepcidin levels along with the improvement of iron absorption [26]. Moreover, other inflammatory factors such as CRP, leptin, and Interleukin-6 have been found to play an important role in the homeostasis of iron [26].

Iron is an essential mineral whose primary function is to transport oxygen in the blood. Inadequate iron status in the form of iron deficiency anemia leads to poor growth and development and the potential for cognitive deficits in children. Thus, children should include food sources of heme iron include red meats, enriched cereal grains, and fortified breakfast cereals.

All nutrients intake with an exception of fiber, magnesium, calcium and vitamin D and vitamin Ε, were shown to be much higher for boys and girls compared with the daily recommended intakes. Special attention should be given to caffeine that was found to be consumed in relatively high levels possible from chocolate or soda drinks. The recommendations for children are almost zero mg while for adolescents up to 100 mg [27]. Sugar levels were clinically higher in obese children. Even though sugar was not independently associated with obesity, it is well-known now that it contributes significantly to pediatric obesity [28].

Energy intake was significantly higher in the overweight/obese group compared to the normal one. The mean number of energy intake in obese children was lower than the usual possibly due to an underreporting issue which is very common in overweight/obese children [29].

In the present study, overweight/obese children consumed a significantly larger portion of their energy from carbohydrates, protein and sugar and lower fiber than their matched controls. Similarly, Eck et al. [30] have shown that obese children consumed a higher percentage of fat energy and from carbohydrate energy compared with normal ones. Moreover, authors have supported the hypothesis that simple sugars have a high contribution to obesity, diabetes type 2 and cardiovascular disease [31]. Additionally, fiber is also related to obesity through various mechanisms. Dietary fiber has the ability to enhance satiety, suppress energy intake and decrease blood glucose levels. By increasing dietary fiber, one might subsequently decrease consumption of other foods high in energy [32].

We also reported that overweight/obese group consumed higher levels of sodium and lower ones of folic acid and zinc. Sodium has been found to positively associate with adiposity in children [33] while low levels of folic acid and zinc were found to relate to cardiovascular disease and obesity by either direct or indirect way [34, 35].

Our study has its limitations. We did not take into account information related to genetics, physical activity, socioeconomic status and values of blood biochemical values. In addition, we did not exclude any outliers for dietary variables. Furthermore, the children were stratified only from three prefectures of Northern Greece and, thus, the number of subjects finally recruited was smaller than initially targeted. This was due to financial reasons that did not allow us to expand the study with a larger number of children. Nevertheless, our data presents very useful information about dietary intakes related to obesity in Greek children emphasizing the need of continuous effort to promote healthy eating habits that must engage all parts of society, such as government, health professionals, mass media, schools and parents in order to prevent and/or treat pediatric obesity.

In conclusion, almost one-third of Greek children of the area of Central Macedonia are overweight or obese. Although most of the dietary intakes in our subjects were adequate, special consideration should be given to energy, carbohydrate, protein, sugar, sodium, and iron intake especially to its relation to obesity. Furthermore, the implication of low dietary iron that was found independently associated with excess weight should be further investigated by additional studies that may possibly reveal a causal effect relation of low dietary iron consumption levels to obesity.
